# Supplementary material for: Influence of honey bee (Apis mellifera) breeding on wing venation in Serbia and neighbouring countries
Source: PeerJ. 2024 Apr 26;12:e17247. doi: 10.7717/peerj.17247 (PMC11057427; doi:10.7717/peerj.17247)
Supplement: Supplemental Information 1 [file peerj-12-17247-s001.html]

Influence of honey bee (Apis mellifera) breeding on wing venation in Serbia and neighbouring countries


# Influence of honey bee (*Apis mellifera*) breeding on wing venation in Serbia and neighbouring countries

Supplementary document with statistical analysis

Authors

Affiliations

Hardeep Kaur

University of Agriculture in Krakow, Poland

Nebojša Nedić

University of Belgrade, Serbia

Adam Tofilski

University of Agriculture in Krakow, Poland

Published

2024-03-13

# Libraries

```
# calculations
library(geomorph)  # GPA 
library(Morpho)  # CVA
library(mgcv)  # GAM
library(mgcViz)  # GAM
library(stats)  # MANOVA
library(phangorn)  # UPGMA
# plotting and visualization
library(ggplot2)  # plots
ggplot2::theme_set(theme_light())
library(rnaturalearth)  # maps
library(raster)  # raster
library(ggspatial)  # annotation_scale
library(viridis)  # colour gradient
```

# Variable names

```
p <- 19  # number of landmarks
k <- 2  # number of dimensions, in this case 2 for coordinates (x, y)

# create coordinates names used by IdentiFly
xyNames <- c("x1", "y1")
for (i in 2:p) {
  xyNames <- c(xyNames, paste0("x", i))
  xyNames <- c(xyNames, paste0("y", i))
}
xyNames
```

```
 [1] "x1"  "y1"  "x2"  "y2"  "x3"  "y3"  "x4"  "y4"  "x5"  "y5"  "x6"  "y6" 
[13] "x7"  "y7"  "x8"  "y8"  "x9"  "y9"  "x10" "y10" "x11" "y11" "x12" "y12"
[25] "x13" "y13" "x14" "y14" "x15" "y15" "x16" "y16" "x17" "y17" "x18" "y18"
[37] "x19" "y19"
```

```
# The number of principal components used is 2*p-4 = 34, which is equal to the
# degrees of freedom create principal components names used by prcomp
pcNames <- paste0("PC", 1:(2 * p - 4))
pcNames
```

```
 [1] "PC1"  "PC2"  "PC3"  "PC4"  "PC5"  "PC6"  "PC7"  "PC8"  "PC9"  "PC10"
[11] "PC11" "PC12" "PC13" "PC14" "PC15" "PC16" "PC17" "PC18" "PC19" "PC20"
[21] "PC21" "PC22" "PC23" "PC24" "PC25" "PC26" "PC27" "PC28" "PC29" "PC30"
[31] "PC31" "PC32" "PC33" "PC34"
```

```
# define which landmarks are connected by lines in wireframe graph
link.x <- c(1, 1, 2, 2, 3, 3, 4, 4, 5, 6, 7, 7, 7, 8, 9, 9, 10, 11, 11, 12, 13, 14,
  15, 16, 17)
link.y <- c(2, 3, 4, 5, 6, 19, 6, 10, 12, 8, 8, 14, 19, 9, 10, 15, 11, 12, 16, 13,
  18, 15, 16, 17, 18)
links.apis <- cbind(link.x, link.y)
```

# Read Serbia data

Some data about honey bees in Serbia originate from an earlier study Oleksa et al., 2022, Oleksa et al., 2023. Data newly obtained for this study is available at Zenodo Kaur et al., 2023

```
wings <- read.csv("https://zenodo.org/record/7244070/files/RS-raw-coordinates.csv")
wings <- rbind(wings, read.csv("https://zenodo.org/record/10389960/files/RS_21_80-raw-coordinates.csv"))
# extract sample classifier from file name
wings$sample <- substr(wings$file, 1, 7)

# Read geographic coordinates
geo.data <- read.csv("https://zenodo.org/record/7244070/files/RS-data.csv")
geo.data <- rbind(geo.data, read.csv("https://zenodo.org/record/10389960/files/RS_21_80-data.csv"))

sample.geo.data <- aggregate(geo.data[c("latitude", "longitude", "date")], by = list(geo.data$sample),
  FUN = mean)
sample.geo.data <- data.frame(sample.geo.data, row.names = 1)  # move column 1 to row names
```

# Read C lineage data

As reference for SE Europe we used the data obtained from the earlier study Oleksa et al., 2022, Oleksa et al., 2023.

```
wings.C <- read.csv("https://zenodo.org/record/7244070/files/EU-raw-coordinates.csv")
geo.data.C <- read.csv("https://zenodo.org/record/7244070/files/EU-geo-data.csv")

wings.C$latitude <- geo.data.C$latitude
wings.C$longitude <- geo.data.C$longitude

wings.C <- subset(wings.C, country %in% c("AT", "GR", "HR", "HU", "MD", "ME", "RO",
  "SI", "TR"))

# GPA Convert 2D array into a 3D array
wings.C.3D <- arrayspecs(wings.C[xyNames], p, k)
dimnames(wings.C.3D)[[3]] <- wings.C$file
# Align the coordinates using Generalized Procrustes Analysis
GPA.C <- gpagen(wings.C.3D, print.progress = FALSE)
# Convert 3D array into a 2D array - opposite to arrayspecs
wings.C.aligned <- two.d.array(GPA.C$coords)

# rename column names from 1.X, 1.Y to x1, y1 ...
colnames(wings.C.aligned) <- gsub("([0-9]+)\\.([XY])", "\\L\\2\\1", colnames(wings.C.aligned),
  perl = TRUE)

# average data within samples
C.samples <- aggregate(wings.C.aligned, by = list(wings.C$sample), FUN = mean)
C.samples <- data.frame(C.samples, row.names = 1)  # move column 1 to row names

# average geo data
geo.sample <- aggregate(wings.C[c("latitude", "longitude")], by = list(wings.C$sample),
  FUN = mean)
geo.sample <- data.frame(geo.sample, row.names = 1)  # move column 1 to row names
C.samples <- cbind(C.samples, geo.sample)
```

# Map

```
# Read elevation data In order to download the TIF file uncomment the two lines
# below
# download.file('https://geodata.ucdavis.edu/climate/worldclim/2_1/base/wc2.1_2.5m_elev.zip',
# 'wc2.1_2.5m_elev.zip') unzip('wc2.1_2.5m_elev.zip') or use your local file
elev <- raster("C:/data/WorldClim/wc2.1_2.5m_elev.tif")
elev.color <- colorRampPalette(c("#f7f7f7", "#f0f0f0", "#d9d9d9", "#bdbdbd", "#636363"),
  bias = 2)

x.min <- 18
x.max <- 24
y.min <- 41.8
y.max <- 46.5
e <- extent(x.min - 1, x.max + 1, y.min - 1, y.max + 1)
elev <- crop(elev, e)
elev.df <- data.frame(rasterToPoints(elev))
colnames(elev.df) <- c("longitude", "latitude", "altitude")
world <- ne_countries(scale = "medium", returnclass = "sf")

# Names of neighboring countries
neighboring_countries <- data.frame(name = c("Croatia", "Hungary", "Romania", "Bulgaria",
  "North Macedonia", "Albania", "Montenegro", "Bosnia and \nHerzegovina", "Kosovo*",
  "Serbia"), latitude = c(45.4, 46.4, 45.44, 42.68, 41.91, 42.1, 42.8, 44, 42.5,
  44.7), longitude = c(18.2, 19.82, 22.5, 23.39, 21.7, 19.82, 19.37, 18.3, 20.8,
  20.4))
# * The designation of Kosovo is without prejudice to positions on status, and
# is in line with UNSCR 1244/1999 and ICJ Opinion on the Kosovo declaration of
# independence.

ggplot(data = world) + geom_raster(data = elev.df, aes(longitude, latitude, fill = altitude)) +
  scale_fill_gradientn(colours = elev.color(100)) + geom_sf(fill = NA) + geom_point(data = sample.geo.data,
  aes(x = longitude, y = latitude, color = as.factor(date))) + scale_color_manual(name = "year",
  values = c("#E69F00", "#009E73", "#56B4E9")) + coord_fixed() + coord_sf(xlim = c(x.min,
  x.max), ylim = c(y.min, y.max)) + geom_text(data = neighboring_countries, aes(x = longitude,
  y = latitude, label = name), color = "black", size = 3, nudge_y = 0.02) + annotation_scale(location = "tr",
  width_hint = 0.2)
```

# GPA alignment

```
# Convert from 2D array to 3D array
wings.coords <- arrayspecs(wings[xyNames], p, k)
dimnames(wings.coords)[[3]] <- wings$file

# Align the coordinates using Generalized Procrustes Analysis
GPA.RS <- gpagen(wings.coords, print.progress = FALSE)

# plot landmarks after alignment
plotAllSpecimens(GPA.RS$coords, links = links.apis, label = TRUE, plot.param = list(pt.bg = "black",
  pt.cex = 0.5, mean.bg = "red", mean.cex = 1, link.col = "red", txt.pos = 3, txt.cex = 1))
```

```
# Convert from 3D array to 2D array
wings.aligned.RS <- two.d.array(GPA.RS$coords)
# rename column names from 1.X, 1.Y to x1, y1 ...
colnames(wings.aligned.RS) <- gsub("([0-9]+)\\.([XY])", "\\L\\2\\1", colnames(wings.aligned.RS),
  perl = TRUE)

# Average coorinates within samples
RS.samples <- aggregate(wings.aligned.RS, by = list(wings$sample), FUN = mean)
RS.samples <- data.frame(RS.samples, row.names = 1)  # move column 1 to row names
RS.samples <- cbind(RS.samples, sample.geo.data)
```

# PCA Serbia - with breeders

```
RS.pca <- prcomp(RS.samples[, xyNames])
RS.pca.scores <- as.data.frame(RS.pca$x[, pcNames])
RS.pca.scores <- cbind(RS.pca.scores, sample.geo.data)

# create plot labels
variance.tab <- summary(RS.pca)$importance
variance <- variance.tab["Proportion of Variance", "PC1"]
variance <- round(100 * variance, 1)
label.x <- paste0("PC1 (", variance, "%)")
variance <- variance.tab["Proportion of Variance", "PC2"]
variance <- round(100 * variance, 1)
label.y <- paste0("PC2 (", variance, "%)")

PCA.A <- ggplot(RS.pca.scores, aes(x = PC1, y = PC2, color = as.factor(date))) +
  geom_point() + scale_color_manual(name = "year", values = c("#E69F00", "#009E73",
  "#56B4E9")) + stat_ellipse() + xlab(label.x) + ylab(label.y) + theme(legend.position = c(0.9,
  0.15), legend.background = element_rect(fill = "white", color = "black"), legend.title = element_blank())

plot(PCA.A)
```

```
# Serbian bees collected in different years differ significantly in wing shape
year.MANOVA <- manova(as.matrix(RS.pca.scores[, pcNames]) ~ date, data = RS.pca.scores)
summary(year.MANOVA)
```

```
          Df  Pillai approx F num Df den Df    Pr(>F)    
date       1 0.79193   5.0374     34     45 3.848e-07 ***
Residuals 78                                             
---
Signif. codes:  0 '***' 0.001 '**' 0.01 '*' 0.05 '.' 0.1 ' ' 1
```

# GAM Serbia - with breeders

```
# PC1
ggplot(data = world) + geom_sf() + geom_point(data = RS.pca.scores, aes(x = longitude,
  y = latitude, colour = PC1), size = 1) + coord_sf(xlim = c(17.4, 24.2), ylim = c(41.8,
  46.5), expand = FALSE) + scale_color_viridis(name = "PC1") + xlab("Longitude") +
  ylab("Latitude")
```

```
GAM <- mgcv::gam(PC1 ~ s(longitude, latitude), data = RS.pca.scores)
anova(GAM)
```

```
Family: gaussian 
Link function: identity 

Formula:
PC1 ~ s(longitude, latitude)

Approximate significance of smooth terms:
                        edf Ref.df     F p-value
s(longitude,latitude) 16.99  21.73 5.286  <2e-16
```

```
viz.GAM <- getViz(GAM)
GAM.A <- plot(viz.GAM, 1) + l_fitRaster() + l_fitContour(color = "grey30") + l_points() +
  labs(title = NULL) + scale_fill_continuous(type = "viridis", name = "PC1", na.value = "transparent") +
  scale_x_continuous(limits = c(17.4, 24.2), expand = c(0, 0)) + scale_y_continuous(limits = c(41.8,
  46.5), expand = c(0, 0)) + geom_path(data = map_data("world"), aes(x = long,
  y = lat, group = group), color = "black", inherit.aes = FALSE)
GAM.A
```

# CVA SE Europe - with breeders

```
C.samples$date <- NA
wings.C.all <- rbind(C.samples, RS.samples)

# GPA Convert 2D array into a 3D array
wings.C.3D <- arrayspecs(wings.C.all[xyNames], p, k)
dimnames(wings.C.3D)[[3]] <- rownames(wings.C.all)
# Align the coordinates using Generalized Procrustes Analysis
GPA.C <- gpagen(wings.C.3D, print.progress = FALSE)
# Convert 3D array into a 2D array - opposite to arrayspecs
wings.C.aligned <- two.d.array(GPA.C$coords)
# rename column names from 1.X, 1.Y to x1, y1 ...
colnames(wings.C.aligned) <- gsub("([0-9]+)\\.([XY])", "\\L\\2\\1", colnames(wings.C.aligned),
  perl = TRUE)

PCA.C <- prcomp(wings.C.aligned[, xyNames])
PCA.C.scores <- as.data.frame(PCA.C$x[, pcNames])
PCA.C.scores$country <- substr(rownames(wings.C.all), 1, 2)

PCA.C.scores$country <- as.factor(PCA.C.scores$country)
levels(PCA.C.scores$country) <- list(Austria = "AT", Croatia = "HR", Greece = "GR",
  Hungary = "HU", Moldova = "MD", Montenegro = "ME", Romania = "RO", Serbia = "RS",
  Slovenia = "SI", Turkey = "TR")

PCA.C.scores <- cbind(PCA.C.scores, wings.C.all[c("latitude", "longitude", "date")])

# create plot labels
variance.tab <- summary(PCA.C)$importance
variance <- variance.tab["Proportion of Variance", "PC1"]
variance <- round(100 * variance, 1)
label.x <- paste0("PC1 (", variance, "%)")
variance <- variance.tab["Proportion of Variance", "PC2"]
variance <- round(100 * variance, 1)
label.y <- paste0("PC2 (", variance, "%)")

ggplot(PCA.C.scores, aes(x = PC1, y = PC2, color = country)) + geom_point() + stat_ellipse()
```

```
# CVA
PCA.C.scores$group.year <- ifelse(PCA.C.scores$country == "Serbia", paste(as.character(PCA.C.scores$country),
  PCA.C.scores$date, sep = "-"), as.character(PCA.C.scores$country))

# use equal prior probability for all groups
n.gr <- length(unique(PCA.C.scores$group.year))  # number of groups
sample.cva <- CVA(PCA.C.scores[pcNames], PCA.C.scores$group.year, rounds = 10000,
  cv = TRUE, prior = rep(1/n.gr, n.gr))
sample.cva.scores <- as.data.frame(sample.cva$CVscores)
# remove unwanted spaces in variable names otherwise use `CV 1`
colnames(sample.cva.scores) <- gsub(" ", "", colnames(sample.cva.scores))
# sample.cva.scores <- cbind(sample.geo.data, sample.cva.scores)
sample.cva.scores$group.year <- PCA.C.scores$group.year

ggplot(sample.cva.scores, aes(x = CV1, y = CV2, shape = group.year, color = group.year)) +
  geom_point() + scale_shape_manual(name = "group", values = c(1:12)) + scale_color_manual(name = "group",
  values = rainbow(12)) + stat_ellipse()
```

```
# Confusion matrix Classification of the samples to groups
CVA.class <- typprobClass(sample.cva$CVscores, groups = as.factor(sample.cva.scores$group.year),
  outlier = 0)
print(CVA.class)
```

```
 cross-validated classification results in frequencies
             
              Austria Croatia Greece Hungary Moldova Montenegro Romania
  Austria          10       0      0       0       0          0       0
  Croatia           0     120      0       7       0          0       0
  Greece            0       0    231       0       0          0       0
  Hungary           0       0      0      17       0          0       5
  Moldova           0       0      0       0       9          0       1
  Montenegro        0       0      0       0       0         17       0
  Romania           0       2      1       2       2          0     183
  Serbia-2005       0       0      0       0       0          0       0
  Serbia-2010       0       0      0       0       0          5       0
  Serbia-2022       0       0      0       0       0          0       0
  Slovenia          0       1      0       2       1          0       0
  Turkey            0       0      0       0       0          0       0
             
              Serbia-2005 Serbia-2010 Serbia-2022 Slovenia Turkey
  Austria               0           0           0        0      0
  Croatia               3           0           6       24      0
  Greece                9           0           1        0      3
  Hungary               0           0           0        0      0
  Moldova               0           0           0        0      0
  Montenegro            0           3           0        0      0
  Romania               0           0           0        7      0
  Serbia-2005           3           0           1        0      0
  Serbia-2010           0          15           0        0      0
  Serbia-2022           1           0          55        0      0
  Slovenia              0           0           0       17      0
  Turkey                0           0           1        0     59


 cross-validated classification result in %
             
                Austria   Croatia    Greece   Hungary   Moldova Montenegro
  Austria     100.00000   0.00000   0.00000   0.00000   0.00000    0.00000
  Croatia       0.00000  75.00000   0.00000   4.37500   0.00000    0.00000
  Greece        0.00000   0.00000  94.67213   0.00000   0.00000    0.00000
  Hungary       0.00000   0.00000   0.00000  77.27273   0.00000    0.00000
  Moldova       0.00000   0.00000   0.00000   0.00000  90.00000    0.00000
  Montenegro    0.00000   0.00000   0.00000   0.00000   0.00000   85.00000
  Romania       0.00000   1.01523   0.50761   1.01523   1.01523    0.00000
  Serbia-2005   0.00000   0.00000   0.00000   0.00000   0.00000    0.00000
  Serbia-2010   0.00000   0.00000   0.00000   0.00000   0.00000   25.00000
  Serbia-2022   0.00000   0.00000   0.00000   0.00000   0.00000    0.00000
  Slovenia      0.00000   4.76190   0.00000   9.52381   4.76190    0.00000
  Turkey        0.00000   0.00000   0.00000   0.00000   0.00000    0.00000
             
                Romania Serbia-2005 Serbia-2010 Serbia-2022  Slovenia    Turkey
  Austria       0.00000     0.00000     0.00000     0.00000   0.00000   0.00000
  Croatia       0.00000     1.87500     0.00000     3.75000  15.00000   0.00000
  Greece        0.00000     3.68852     0.00000     0.40984   0.00000   1.22951
  Hungary      22.72727     0.00000     0.00000     0.00000   0.00000   0.00000
  Moldova      10.00000     0.00000     0.00000     0.00000   0.00000   0.00000
  Montenegro    0.00000     0.00000    15.00000     0.00000   0.00000   0.00000
  Romania      92.89340     0.00000     0.00000     0.00000   3.55330   0.00000
  Serbia-2005   0.00000    75.00000     0.00000    25.00000   0.00000   0.00000
  Serbia-2010   0.00000     0.00000    75.00000     0.00000   0.00000   0.00000
  Serbia-2022   0.00000     1.78571     0.00000    98.21429   0.00000   0.00000
  Slovenia      0.00000     0.00000     0.00000     0.00000  80.95238   0.00000
  Turkey        0.00000     0.00000     0.00000     1.66667   0.00000  98.33333


 overall classification accuracy: 89.32039 %

 Kappa statistic: 0.86945
```

```
# Mahalanobis distances between groups
knitr::kable(as.data.frame(as.matrix(sample.cva$Dist$GroupdistMaha)), digits = 3)
```

|  | Austria | Croatia | Greece | Hungary | Moldova | Montenegro | Romania | Serbia-2005 | Serbia-2010 | Serbia-2022 | Slovenia | Turkey |
| --- | --- | --- | --- | --- | --- | --- | --- | --- | --- | --- | --- | --- |
| Austria | 0.000 | 7.997 | 9.919 | 6.901 | 8.242 | 8.055 | 6.947 | 8.225 | 8.036 | 7.695 | 8.843 | 8.324 |
| Croatia | 7.997 | 0.000 | 5.703 | 3.604 | 5.694 | 5.992 | 4.264 | 3.591 | 5.257 | 3.766 | 2.732 | 5.224 |
| Greece | 9.919 | 5.703 | 0.000 | 7.300 | 7.803 | 6.868 | 6.345 | 4.735 | 6.682 | 5.490 | 6.468 | 5.648 |
| Hungary | 6.901 | 3.604 | 7.300 | 0.000 | 5.990 | 6.944 | 4.198 | 5.637 | 6.342 | 5.270 | 4.306 | 6.263 |
| Moldova | 8.242 | 5.694 | 7.803 | 5.990 | 0.000 | 7.288 | 4.253 | 6.173 | 6.597 | 6.151 | 5.428 | 7.535 |
| Montenegro | 8.055 | 5.992 | 6.868 | 6.944 | 7.288 | 0.000 | 5.732 | 6.009 | 2.085 | 5.059 | 7.265 | 6.945 |
| Romania | 6.947 | 4.264 | 6.345 | 4.198 | 4.253 | 5.732 | 0.000 | 5.201 | 5.269 | 5.146 | 4.703 | 5.402 |
| Serbia-2005 | 8.225 | 3.591 | 4.735 | 5.637 | 6.173 | 6.009 | 5.201 | 0.000 | 5.746 | 3.161 | 4.697 | 5.101 |
| Serbia-2010 | 8.036 | 5.257 | 6.682 | 6.342 | 6.597 | 2.085 | 5.269 | 5.746 | 0.000 | 5.039 | 6.469 | 7.021 |
| Serbia-2022 | 7.695 | 3.766 | 5.490 | 5.270 | 6.151 | 5.059 | 5.146 | 3.161 | 5.039 | 0.000 | 4.916 | 5.742 |
| Slovenia | 8.843 | 2.732 | 6.468 | 4.306 | 5.428 | 7.265 | 4.703 | 4.697 | 6.469 | 4.916 | 0.000 | 6.320 |
| Turkey | 8.324 | 5.224 | 5.648 | 6.263 | 7.535 | 6.945 | 5.402 | 5.101 | 7.021 | 5.742 | 6.320 | 0.000 |

```
# pairwise probabilities of differences between groups
knitr::kable(as.data.frame(as.matrix(sample.cva$Dist$probsMaha)), digits = 5)
```

|  | Austria | Croatia | Greece | Hungary | Moldova | Montenegro | Romania | Serbia-2005 | Serbia-2010 | Serbia-2022 | Slovenia | Turkey |
| --- | --- | --- | --- | --- | --- | --- | --- | --- | --- | --- | --- | --- |
| Austria | 0e+00 | 0.00010 | 0.0001 | 0.0001 | 0.0001 | 0.00010 | 0.0001 | 0.00010 | 0.00010 | 0.00010 | 0.00010 | 0.0001 |
| Croatia | 1e-04 | 0.00000 | 0.0001 | 0.0001 | 0.0001 | 0.00010 | 0.0001 | 0.33477 | 0.00010 | 0.00010 | 0.00080 | 0.0001 |
| Greece | 1e-04 | 0.00010 | 0.0000 | 0.0001 | 0.0001 | 0.00010 | 0.0001 | 0.02040 | 0.00010 | 0.00010 | 0.00010 | 0.0001 |
| Hungary | 1e-04 | 0.00010 | 0.0001 | 0.0000 | 0.0001 | 0.00010 | 0.0001 | 0.00440 | 0.00010 | 0.00010 | 0.00010 | 0.0001 |
| Moldova | 1e-04 | 0.00010 | 0.0001 | 0.0001 | 0.0000 | 0.00010 | 0.0001 | 0.00360 | 0.00010 | 0.00010 | 0.00010 | 0.0001 |
| Montenegro | 1e-04 | 0.00010 | 0.0001 | 0.0001 | 0.0001 | 0.00000 | 0.0001 | 0.00180 | 0.52105 | 0.00010 | 0.00010 | 0.0001 |
| Romania | 1e-04 | 0.00010 | 0.0001 | 0.0001 | 0.0001 | 0.00010 | 0.0000 | 0.00510 | 0.00010 | 0.00010 | 0.00010 | 0.0001 |
| Serbia-2005 | 1e-04 | 0.33477 | 0.0204 | 0.0044 | 0.0036 | 0.00180 | 0.0051 | 0.00000 | 0.00340 | 0.69503 | 0.05249 | 0.0091 |
| Serbia-2010 | 1e-04 | 0.00010 | 0.0001 | 0.0001 | 0.0001 | 0.52105 | 0.0001 | 0.00340 | 0.00000 | 0.00010 | 0.00010 | 0.0001 |
| Serbia-2022 | 1e-04 | 0.00010 | 0.0001 | 0.0001 | 0.0001 | 0.00010 | 0.0001 | 0.69503 | 0.00010 | 0.00000 | 0.00010 | 0.0001 |
| Slovenia | 1e-04 | 0.00080 | 0.0001 | 0.0001 | 0.0001 | 0.00010 | 0.0001 | 0.05249 | 0.00010 | 0.00010 | 0.00000 | 0.0001 |
| Turkey | 1e-04 | 0.00010 | 0.0001 | 0.0001 | 0.0001 | 0.00010 | 0.0001 | 0.00910 | 0.00010 | 0.00010 | 0.00010 | 0.0000 |

# UPGMA - with breeders

```
UPBMA.A <- upgma(sample.cva$Dist$GroupdistMaha)
plot(UPBMA.A, label.offset = 0.1, cex = 0.5)
```

# Remove breeders

```
RS.samples.short <- subset(RS.samples, date %in% c("2005", "2022"))
```

# PCA Serbia - without breeders

```
RS.pca <- prcomp(RS.samples.short[, xyNames])
RS.pca.scores <- as.data.frame(RS.pca$x[, pcNames])
RS.pca.scores$latitude <- RS.samples.short$latitude
RS.pca.scores$longitude <- RS.samples.short$longitude
RS.pca.scores$date <- RS.samples.short$date

# create plot labels
variance.tab <- summary(RS.pca)$importance
variance <- variance.tab["Proportion of Variance", "PC1"]
variance <- round(100 * variance, 1)
label.x <- paste0("PC1 (", variance, "%)")
variance <- variance.tab["Proportion of Variance", "PC2"]
variance <- round(100 * variance, 1)
label.y <- paste0("PC2 (", variance, "%)")

PCA.B <- ggplot(RS.pca.scores, aes(x = PC1, y = PC2, color = as.factor(date))) +
  geom_point() + scale_color_manual(name = "year", values = c("#E69F00", "#56B4E9")) +
  stat_ellipse() + xlab(label.x) + ylab(label.y) + scale_x_continuous(expand = c(0.06,
  0)) + theme(legend.position = c(0.9, 0.15), legend.background = element_rect(fill = "white",
  color = "black"), legend.title = element_blank())

plot(PCA.B)
```

# GAM Serbia - without breeders

```
# PC1
ggplot(data = world) + geom_sf() + geom_point(data = RS.pca.scores, aes(x = longitude,
  y = latitude, colour = PC1), size = 1) + coord_sf(xlim = c(17.4, 24.2), ylim = c(41.8,
  46.5), expand = FALSE) + scale_color_viridis(name = "PC1") + xlab("Longitude") +
  ylab("Latitude")
```

```
GAM <- mgcv::gam(PC1 ~ s(longitude, latitude), data = RS.pca.scores)
anova(GAM)
```

```
Family: gaussian 
Link function: identity 

Formula:
PC1 ~ s(longitude, latitude)

Approximate significance of smooth terms:
                        edf Ref.df     F p-value
s(longitude,latitude) 3.844  5.170 2.068  0.0812
```

```
viz.GAM <- getViz(GAM)
GAM.B <- plot(viz.GAM, 1) + l_fitRaster() + l_fitContour(color = "grey30") + l_points() +
  labs(title = NULL) + scale_fill_continuous(type = "viridis", name = "PC1", na.value = "transparent") +
  scale_x_continuous(limits = c(17.4, 24.2), expand = c(0, 0)) + scale_y_continuous(limits = c(41.8,
  46.5), expand = c(0, 0)) + geom_path(data = map_data("world"), aes(x = long,
  y = lat, group = group), color = "black", inherit.aes = FALSE)
GAM.B
```

# PCA SE Europe - without breeders

```
countries <- substr(rownames(C.samples), 1, 2)
C.samples.short <- subset(C.samples, countries %in% c("GR", "HR", "HU", "MD", "RO",
  "SI", "TR"))
samples <- rbind(C.samples.short, RS.samples.short)

# GPA Convert 2D array into a 3D array
samples.3D <- arrayspecs(samples[xyNames], p, k)
dimnames(samples.3D)[[3]] <- rownames(samples)
# Align the coordinates using Generalized Procrustes Analysis
GPA.C <- gpagen(samples.3D, print.progress = FALSE)
# Convert 3D array into a 2D array - opposite to arrayspecs
samples.aligned <- two.d.array(GPA.C$coords)
# rename column names from 1.X, 1.Y to x1, y1 ...
colnames(samples.aligned) <- gsub("([0-9]+)\\.([XY])", "\\L\\2\\1", colnames(samples.aligned),
  perl = TRUE)

# PCA
PCA.C <- prcomp(samples.aligned[, xyNames])
PCA.C.scores <- as.data.frame(PCA.C$x[, pcNames])
# change sign of the PC1 for easier comparisons with earlier graphs
PCA.C.scores[, "PC1"] <- -PCA.C.scores[, "PC1"]
PCA.C.scores$latitude <- samples$latitude[]
PCA.C.scores$longitude <- samples$longitude[]

PCA.C.scores$country <- substr(rownames(samples), 1, 2)
PCA.C.scores$country <- as.factor(PCA.C.scores$country)
levels(PCA.C.scores$country) <- list(Croatia = "HR", Greece = "GR", Hungary = "HU",
  Moldova = "MD", Romania = "RO", Serbia = "RS", Slovenia = "SI", Turkey = "TR")

# create plot labels
variance.tab <- summary(PCA.C)$importance
variance <- variance.tab["Proportion of Variance", "PC1"]
variance <- round(100 * variance, 1)
label.x <- paste0("PC1 (", variance, "%)")
variance <- variance.tab["Proportion of Variance", "PC2"]
variance <- round(100 * variance, 1)
label.y <- paste0("PC2 (", variance, "%)")

ggplot(PCA.C.scores, aes(x = PC1, y = PC2, color = country)) + geom_point() + stat_ellipse()
```

```
# PC1
ggplot(data = world) + geom_sf() + geom_point(data = PCA.C.scores, aes(x = longitude,
  y = latitude, colour = PC1), size = 1) + coord_sf(xlim = c(10, 30), ylim = c(33,
  50), expand = FALSE) + scale_color_viridis(name = "PC1") + xlab("Longitude") +
  ylab("Latitude")
```

```
GAM <- mgcv::gam(PC1 ~ s(longitude, latitude), data = PCA.C.scores)
anova(GAM)
```

```
Family: gaussian 
Link function: identity 

Formula:
PC1 ~ s(longitude, latitude)

Approximate significance of smooth terms:
                        edf Ref.df     F p-value
s(longitude,latitude) 23.09  26.94 83.45  <2e-16
```

```
viz.GAM <- getViz(GAM)
plot(viz.GAM, 1) + l_fitRaster() + l_fitContour(color = "grey30") + l_points() +
  labs(title = NULL) + scale_fill_continuous(type = "viridis", name = "PC1", na.value = "transparent") +
  scale_x_continuous(limits = c(10, 30), expand = c(0, 0)) + scale_y_continuous(limits = c(33,
  50), expand = c(0, 0)) + geom_path(data = map_data("world"), aes(x = long, y = lat,
  group = group), color = "black", inherit.aes = FALSE)
```

```
# PC2
ggplot(data = world) + geom_sf() + geom_point(data = PCA.C.scores, aes(x = longitude,
  y = latitude, colour = PC2), size = 1) + coord_sf(xlim = c(10, 30), ylim = c(33,
  50), expand = FALSE) + scale_color_viridis(name = "PC1") + xlab("Longitude") +
  ylab("Latitude")
```

```
GAM <- mgcv::gam(PC2 ~ s(longitude, latitude), data = PCA.C.scores)
anova(GAM)
```

```
Family: gaussian 
Link function: identity 

Formula:
PC2 ~ s(longitude, latitude)

Approximate significance of smooth terms:
                        edf Ref.df     F p-value
s(longitude,latitude) 25.27  28.13 29.35  <2e-16
```

```
viz.GAM <- getViz(GAM)
plot(viz.GAM, 1) + l_fitRaster() + l_fitContour(color = "grey30") + l_points() +
  labs(title = NULL) + scale_fill_continuous(type = "viridis", name = "PC2", na.value = "transparent") +
  scale_x_continuous(limits = c(10, 30), expand = c(0, 0)) + scale_y_continuous(limits = c(33,
  50), expand = c(0, 0)) + geom_path(data = map_data("world"), aes(x = long, y = lat,
  group = group), color = "black", inherit.aes = FALSE)
```

# CVA SE Europe - without breeders

```
# use equal prior probability for all groups
n.gr <- length(unique(PCA.C.scores$country))  # number of groups
sample.cva <- CVA(PCA.C.scores[pcNames], PCA.C.scores$country, rounds = 10000, cv = TRUE,
  prior = rep(1/n.gr, n.gr))
sample.cva.scores <- as.data.frame(sample.cva$CVscores)
# remove unwanted spaces in variable names otherwise use `CV 1`
colnames(sample.cva.scores) <- gsub(" ", "", colnames(sample.cva.scores))
sample.cva.scores$country <- PCA.C.scores$country

ggplot(sample.cva.scores, aes(x = CV1, y = CV2, shape = country, color = country)) +
  geom_point() + scale_shape_manual(name = "country", values = c(1:8)) + scale_color_manual(name = "country",
  values = rainbow(8)) + stat_ellipse()
```

```
# Confusion matrix Classification of the samples to groups
CVA.class <- typprobClass(sample.cva$CVscores, groups = as.factor(sample.cva.scores$country),
  outlier = 0)
print(CVA.class)
```

```
 cross-validated classification results in frequencies
          
           Croatia Greece Hungary Moldova Romania Serbia Slovenia Turkey
  Croatia      123      1       7       0       0      5       24      0
  Greece         1    238       0       0       0      3        0      2
  Hungary        0      0      17       0       5      0        0      0
  Moldova        0      0       0       9       1      0        0      0
  Romania        2      1       2       2     183      0        7      0
  Serbia         1      0       0       0       0     59        0      0
  Slovenia       1      0       0       1       0      0       19      0
  Turkey         0      0       0       0       0      1        0     59


 cross-validated classification result in %
          
            Croatia   Greece  Hungary  Moldova  Romania   Serbia Slovenia
  Croatia  76.87500  0.62500  4.37500  0.00000  0.00000  3.12500 15.00000
  Greece    0.40984 97.54098  0.00000  0.00000  0.00000  1.22951  0.00000
  Hungary   0.00000  0.00000 77.27273  0.00000 22.72727  0.00000  0.00000
  Moldova   0.00000  0.00000  0.00000 90.00000 10.00000  0.00000  0.00000
  Romania   1.01523  0.50761  1.01523  1.01523 92.89340  0.00000  3.55330
  Serbia    1.66667  0.00000  0.00000  0.00000  0.00000 98.33333  0.00000
  Slovenia  4.76190  0.00000  0.00000  4.76190  0.00000  0.00000 90.47619
  Turkey    0.00000  0.00000  0.00000  0.00000  0.00000  1.66667  0.00000
          
             Turkey
  Croatia   0.00000
  Greece    0.81967
  Hungary   0.00000
  Moldova   0.00000
  Romania   0.00000
  Serbia    0.00000
  Slovenia  0.00000
  Turkey   98.33333


 overall classification accuracy: 91.34367 %

 Kappa statistic: 0.89044
```

```
# Mahalanobis distances between groups
knitr::kable(as.data.frame(as.matrix(sample.cva$Dist$GroupdistMaha)), digits = 3)
```

|  | Croatia | Greece | Hungary | Moldova | Romania | Serbia | Slovenia | Turkey |
| --- | --- | --- | --- | --- | --- | --- | --- | --- |
| Croatia | 0.000 | 5.615 | 3.554 | 5.662 | 4.225 | 3.675 | 2.720 | 5.197 |
| Greece | 5.615 | 0.000 | 7.175 | 7.689 | 6.234 | 5.312 | 6.390 | 5.610 |
| Hungary | 3.554 | 7.175 | 0.000 | 5.958 | 4.157 | 5.186 | 4.275 | 6.196 |
| Moldova | 5.662 | 7.689 | 5.958 | 0.000 | 4.249 | 6.033 | 5.427 | 7.463 |
| Romania | 4.225 | 6.234 | 4.157 | 4.249 | 0.000 | 5.029 | 4.704 | 5.323 |
| Serbia | 3.675 | 5.312 | 5.186 | 6.033 | 5.029 | 0.000 | 4.876 | 5.555 |
| Slovenia | 2.720 | 6.390 | 4.275 | 5.427 | 4.704 | 4.876 | 0.000 | 6.322 |
| Turkey | 5.197 | 5.610 | 6.196 | 7.463 | 5.323 | 5.555 | 6.322 | 0.000 |

```
# pairwise probabilities of differences between groups
knitr::kable(as.data.frame(as.matrix(sample.cva$Dist$probsMaha)), digits = 5)
```

|  | Croatia | Greece | Hungary | Moldova | Romania | Serbia | Slovenia | Turkey |
| --- | --- | --- | --- | --- | --- | --- | --- | --- |
| Croatia | 0e+00 | 1e-04 | 1e-04 | 1e-04 | 1e-04 | 1e-04 | 2e-04 | 1e-04 |
| Greece | 1e-04 | 0e+00 | 1e-04 | 1e-04 | 1e-04 | 1e-04 | 1e-04 | 1e-04 |
| Hungary | 1e-04 | 1e-04 | 0e+00 | 1e-04 | 1e-04 | 1e-04 | 1e-04 | 1e-04 |
| Moldova | 1e-04 | 1e-04 | 1e-04 | 0e+00 | 1e-04 | 1e-04 | 1e-04 | 1e-04 |
| Romania | 1e-04 | 1e-04 | 1e-04 | 1e-04 | 0e+00 | 1e-04 | 1e-04 | 1e-04 |
| Serbia | 1e-04 | 1e-04 | 1e-04 | 1e-04 | 1e-04 | 0e+00 | 1e-04 | 1e-04 |
| Slovenia | 2e-04 | 1e-04 | 1e-04 | 1e-04 | 1e-04 | 1e-04 | 0e+00 | 1e-04 |
| Turkey | 1e-04 | 1e-04 | 1e-04 | 1e-04 | 1e-04 | 1e-04 | 1e-04 | 0e+00 |

# UPGMA - without breeders

```
UPBMA.B <- upgma(sample.cva$Dist$GroupdistMaha)
plot(UPBMA.B, label.offset = 0.1, cex = 0.5)
```

# Figures

```
library(phytools)  # cophylo

cophylo.upgma <- cophylo(UPBMA.A, UPBMA.B, rotate = T)
```

```
Rotating nodes to optimize matching...
Done.
```

```
plot(cophylo.upgma)
```

```
library(ggpubr)  # ggarange
ggarrange(PCA.A, PCA.B, labels = c("A", "B"), font.label = list(size = 12, face = "plain"),
  ncol = 2, nrow = 1)
```

# References

Kaur, H., Nedić, N., & Tofilski, A. (2023). Fore wing images of honey bees (*Apis mellifera*) from Serbia [Data set]. Zenodo. https://doi.org/10.5281/zenodo.10389960

Oleksa, A., Căuia, E., Siceanu, A., Puškadija, Z., Kovačić, M., Pinto, M.A., Rodrigues, P.J., Hatjina, F., Charistos, L., Bouga, M., Prešern, J., Kandemir, I., Rašić, S., Kusza, S., & Tofilski, A. (2022). Collection of wing images for conservation of honey bees (*Apis mellifera*) biodiversity in Europe [Data set]. Zenodo. https://doi.org/10.5281/zenodo.7244070

Oleksa, A., Căuia, E., Siceanu, A., Puškadija, Z., Kovačić, M., Pinto, M. A., Rodrigues, P. J., Hatjina, F., Charistos, L., Bouga, M., Prešern, J., Kandemir, İ., Rašić, S., Kusza, S., Tofilski, A. (2023). Honey bee (*Apis mellifera*) wing images: a tool for identification and conservation. GigaScience, 12, giad019. https://doi.org/10.1093/gigascience/giad019

# Information about session

```
sessionInfo()
```

```
R version 4.3.2 (2023-10-31 ucrt)
Platform: x86_64-w64-mingw32/x64 (64-bit)
Running under: Windows 11 x64 (build 22000)

Matrix products: default


locale:
[1] LC_COLLATE=Polish_Poland.utf8  LC_CTYPE=Polish_Poland.utf8   
[3] LC_MONETARY=Polish_Poland.utf8 LC_NUMERIC=C                  
[5] LC_TIME=Polish_Poland.utf8    

time zone: Europe/Warsaw
tzcode source: internal

attached base packages:
[1] stats     graphics  grDevices utils     datasets  methods   base     

other attached packages:
 [1] ggpubr_0.6.0        phytools_2.0-3      maps_3.4.1.1       
 [4] viridis_0.6.4       viridisLite_0.4.2   ggspatial_1.1.9    
 [7] raster_3.6-26       sp_2.1-1            rnaturalearth_0.3.4
[10] phangorn_2.11.1     ape_5.7-1           mgcViz_0.1.11      
[13] ggplot2_3.4.4       qgam_1.3.4          mgcv_1.9-0         
[16] nlme_3.1-163        Morpho_2.11         geomorph_4.0.6     
[19] Matrix_1.6-1.1      rgl_1.2.1           RRPP_1.4.0         

loaded via a namespace (and not attached):
  [1] RColorBrewer_1.1-3      rstudioapi_0.15.0       jsonlite_1.8.7         
  [4] magrittr_2.0.3          farver_2.1.1            nloptr_2.0.3           
  [7] rmarkdown_2.25          vctrs_0.6.4             minqa_1.2.6            
 [10] base64enc_0.1-3         terra_1.7-55            rstatix_0.7.2          
 [13] htmltools_0.5.7         broom_1.0.5             KernSmooth_2.23-22     
 [16] htmlwidgets_1.6.3       plyr_1.8.9              igraph_1.5.1           
 [19] mime_0.12               lifecycle_1.0.4         iterators_1.0.14       
 [22] pkgconfig_2.0.3         R6_2.5.1                fastmap_1.1.1          
 [25] shiny_1.8.0             digest_0.6.33           numDeriv_2016.8-1.1    
 [28] colorspace_2.1-0        GGally_2.2.0            bezier_1.1.2           
 [31] labeling_0.4.3          clusterGeneration_1.3.8 fansi_1.0.5            
 [34] colorRamps_2.3.1        httr_1.4.7              abind_1.4-5            
 [37] compiler_4.3.2          proxy_0.4-27            withr_2.5.2            
 [40] doParallel_1.0.17       backports_1.4.1         optimParallel_1.0-2    
 [43] carData_3.0-5           DBI_1.1.3               gamm4_0.2-6            
 [46] ggstats_0.5.1           ggsignif_0.6.4          MASS_7.3-60            
 [49] classInt_0.4-10         scatterplot3d_0.3-44    tools_4.3.2            
 [52] units_0.8-4             httpuv_1.6.12           glue_1.6.2             
 [55] rnaturalearthdata_0.1.0 quadprog_1.5-8          promises_1.2.1         
 [58] grid_4.3.2              sf_1.0-14               generics_0.1.3         
 [61] isoband_0.2.7           gtable_0.3.4            Rvcg_0.22.1            
 [64] class_7.3-22            tidyr_1.3.0             car_3.1-2              
 [67] utf8_1.2.4              foreach_1.5.2           pillar_1.9.0           
 [70] later_1.3.1             splines_4.3.2           dplyr_1.1.4            
 [73] lattice_0.21-9          tidyselect_1.2.0        miniUI_0.1.1.1         
 [76] knitr_1.45              gridExtra_2.3           xfun_0.41              
 [79] expm_0.999-8            matrixStats_1.1.0       yaml_2.3.7             
 [82] boot_1.3-28.1           evaluate_0.23           codetools_0.2-19       
 [85] tibble_3.2.1            cli_3.6.1               xtable_1.8-4           
 [88] munsell_0.5.0           Rcpp_1.0.11             coda_0.19-4            
 [91] parallel_4.3.2          ellipsis_0.3.2          jpeg_0.1-10            
 [94] lme4_1.1-35.1           scales_1.2.1            e1071_1.7-13           
 [97] purrr_1.0.2             combinat_0.0-8          rlang_1.1.2            
[100] fastmatch_1.1-4         cowplot_1.1.1           formatR_1.14           
[103] mnormt_2.1.1
```
